# Evaluating the Role of Chemotherapy in Addition to Radiotherapy for High-Risk Merkel Cell Carcinoma

**DOI:** 10.3390/medsci14020311

**Published:** 2026-06-12

**Authors:** Ronen Brenner, Hanna T. Frumin Edri, Amichay Meirovitz, Sabri El-Saied, Keren Rouvinov, Ilia Berezhnov, Anna Ievko, Sofiia Turaieva, Shlomit Fenig, Nashat Abu Yasin, Eyal Fenig, Samer Hussany, Noa Shani Shrem, Alexander Yakobson, Walid Shalata

**Affiliations:** 1 Oncology Institute, Edith Wolfson Medical Center, Holon 58220, Israel; 2 Faculty of Medicine, Tel Aviv University, Tel Aviv 69978, Israel; 3 The Legacy Heritage Cancer Center and Dr. Larry Norton Institute, Soroka Medical Center, Beer Sheva 84105, Israel; 4 Faculty of Health Sciences, Ben Gurion University of the Negev, Beer Sheva 84105, Israel; 5 Institute of Oncology, Kaplan Medical Center, Faculty of Medicine, Hebrew University, Jerusalem 91905, Israel; 6 Institute of Oncology, Davidoff Center, Rabin Medical Center, Beilinson Hospital, Petah Tikva 49414, Israel

**Keywords:** adjuvant therapy, chemoradiotherapy, radiotherapy, high-risk carcinoma, tumor sun-exposure site

## Abstract

Background: Merkel cell carcinoma (MCC) is an aggressive cutaneous neuroendocrine malignancy with a high risk of recurrence. While adjuvant radiotherapy is standard following surgical resection in high-risk disease, the additional benefit of platinum–etoposide chemotherapy and the prognostic role of tumor anatomical location remain uncertain. Methods: We conducted a multicenter retrospective cohort study including patients with high-risk MCC (stage IIB–III) treated with surgery followed by adjuvant radiotherapy with or without platinum–etoposide chemotherapy. Tumor sites were classified according to sun-exposure status. Disease-free survival (DFS) and overall survival (OS) were estimated using Kaplan–Meier methods and compared using the log-rank test, with subgroup analyses by anatomical region and stage. Results: A total of 103 patients were included, of whom 77 (74.8%) received adjuvant chemoradiotherapy and 26 (25.2%) received radiotherapy alone. Patients with non-sun-exposed tumors demonstrated longer survival outcomes than those with sun-exposed tumors, with a median DFS of 57 months versus 42 months (*p* = 0.15), and a median OS of 179 months versus 109 months (*p* = 0.054), respectively. Among patients with sun-exposed tumors, chemoradiotherapy was associated with numerically improved DFS (42 vs. 34 months; *p* = 0.051) and OS (128 vs. 98 months; *p* = 0.08) compared with radiotherapy alone. In patients with non-sun-exposed tumors, chemoradiotherapy demonstrated a more pronounced improvement in OS (178 vs. 56 months; *p* = 0.054), while DFS also favored combined treatment (49 vs. 78 months; *p* = 0.078). Conclusions: In this multicenter cohort, adjuvant chemotherapy did not demonstrate a uniform survival benefit overall but was associated with improved outcomes in head and neck MCC, suggesting a potential site-specific effect. Similar outcomes across stage III subgroups suggest that chemotherapy may mitigate stage-related prognostic differences. These findings support a selective approach to adjuvant chemotherapy and highlight the need for prospective studies incorporating modern immunotherapy strategies.

## 1. Introduction

Merkel cell carcinoma (MCC) is a rare but highly aggressive cutaneous neuroendocrine malignancy characterized by a high propensity for local recurrence, regional nodal involvement, and distant metastasis. Although MCC accounts for less than 1% of all skin cancers, it is responsible for a disproportionate number of skin cancer-related deaths, with reported disease-specific mortality rates ranging from approximately 30% to 40%, exceeding those of melanoma in stage-matched comparisons [1,2]. The incidence of MCC has increased steadily over recent decades, with current estimates in the United States ranging from approximately 0.7 to 1.0 cases per 100,000 persons annually, corresponding to more than 3000 new cases each year [2,3]. This rise has been attributed to an aging population, increased ultraviolet (UV) exposure, greater use of immunosuppressive therapies, and improved diagnostic awareness [1,2,3].

Several risk factors have been associated with the development of MCC, including advanced age, immunosuppression, and chronic exposure to ultraviolet radiation [3]. Consequently, MCC most commonly arises in sun-exposed anatomical sites, particularly the head and neck region, upper extremities, and lower extremities. In contrast, tumors occurring in non-sun-exposed areas such as the trunk, buttock, groin, or thigh have been less frequently reported but may reflect different biological or etiological mechanisms [4]. Previous studies have suggested that tumor location and sun exposure may influence disease behavior and prognosis [5,6].

The standard management of localized MCC typically involves wide local excision followed by adjuvant radiotherapy, particularly in patients with high-risk features such as large tumor size, lymphovascular invasion, positive margins, or nodal involvement [7,8,9]. Adjuvant radiotherapy has been shown to improve local and regional control and is widely recommended in clinical practice guidelines [10,11]. However, the role of adjuvant chemotherapy in addition to radiotherapy remains controversial. While some retrospective studies have suggested that combined chemoradiotherapy may improve disease control in high-risk patients, other reports have failed to demonstrate a significant survival benefit and have raised concerns regarding treatment-related toxicity [12,13].

Furthermore, the impact of tumor sun-exposure status on outcomes following adjuvant treatment strategies has not been well characterized. Given the potential biological differences between MCC arising in sun-exposed versus non-sun-exposed sites, it remains unclear whether treatment response and recurrence risk may vary according to tumor location.

Therefore, the aim of the present retrospective study was to evaluate the clinical outcomes of patients with high-risk MCC treated with adjuvant chemoradiotherapy compared with radiotherapy alone, and to explore whether tumor location according to sun exposure is associated with differences in recurrence and survival outcomes.

## 2. Materials and Methods

### 2.1. The Patient Population and the Design of the Study

Patients with high-risk localized or locally advanced Merkel cell carcinoma treated across multiple institutions were retrospectively reviewed. All individuals underwent surgical resection and subsequently received adjuvant radiotherapy, either as a single modality or in combination with chemotherapy. The primary aim of this study was to evaluate whether the addition of chemotherapy to adjuvant radiotherapy improves clinical outcomes in patients at high risk of recurrence. Patients treated between September 1985 and February 2021 were identified from oncology and pathology databases at several medical centers in Israel (Wolfson Medical Center, Soroka Medical Center, Rabin Medical Center). High-risk disease was defined as pathological T4 tumors without lymph node involvement (stage IIB) or tumors of any T stage with regional lymph node involvement and no evidence of distant metastases (M0), (stage III). Patients were categorized into two groups according to the adjuvant treatment received: those treated with adjuvant chemoradiotherapy consisting of platinum–etoposide-based chemotherapy combined with radiotherapy, and those treated with adjuvant radiotherapy alone without chemotherapy.

### 2.2. Collection of Data

**Figure 1:**
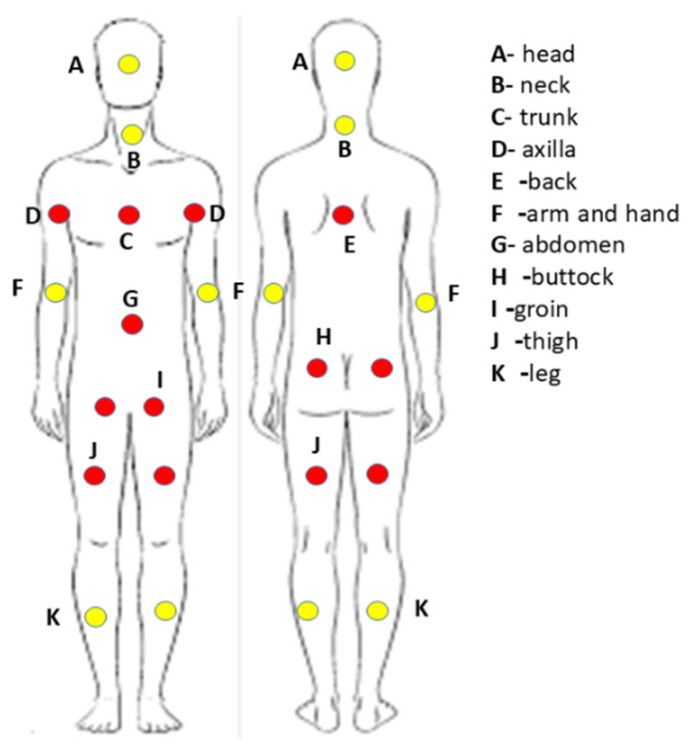
The classification of the sun-exposed sites primarily included the head and neck, lips, arm and hand, and leg (yellow points), whereas non-sun-exposed sites mainly involved chest, trunk, back, abdomen, buttock, groin and thigh (red points).

Potentially eligible cases were retrieved from pathology archives and oncology registries at participating institutions using established diagnostic classification codes. Relevant clinical and histopathological information was obtained from electronic health records and systematically recorded in a dedicated study database by trained research personnel. Collected variables included demographic characteristics such as age at diagnosis and sex, as well as tumor-related variables including primary tumor location and pathological stage according to the TNM classification. Tumor locations were further categorized according to sun exposure status. Sun-exposed sites included the head and neck, lips, arms, hands, and legs, whereas non-sun-exposed sites included the chest, trunk, back, abdomen, buttock, groin, and thigh as in (Figure 1). Treatment-related variables included surgical management, radiotherapy technique and dose, and administration of chemotherapy with the number of cycles delivered. Disease staging at diagnosis was determined according to the American Joint Committee on Cancer (AJCC) TNM staging system, which classifies tumors based on primary tumor characteristics, regional nodal involvement, and distant metastatic spread. Survival outcomes and vital status were obtained from the national population registry maintained by the Israel Ministry of Interior, with follow-up data available through May 2025.

### 2.3. The Inclusion and Exclusion Criteria of Patients

Inclusion criteria comprised adults (≥18 years) with histopathologically verified MCC and high-risk locoregional disease. High-risk status was assigned to patients with T4 tumors without nodal involvement or to those with nodal metastases at any T stage in the absence of distant spread. In addition, all patients must have undergone surgical resection followed by adjuvant radiotherapy with or without chemotherapy as part of their initial treatment strategy and have available clinical follow-up data allowing assessment of survival outcomes. A consecutive sampling approach was applied, whereby all patients meeting the predefined inclusion criteria during the study period were systematically identified and included, provided that sufficient clinical and follow-up data were available. No selective sampling was performed beyond the predefined exclusion criteria. Patients were excluded if they had distant metastatic disease at diagnosis, had pathological stages lower than the predefined high-risk categories, had received prior systemic therapy or radiotherapy for MCC before the index treatment, had a diagnosis other than MCC, or lacked sufficient clinical information to determine the overall survival (OS) or disease-free survival (DFS) and patients with MCC of unknown origin were excluded.

### 2.4. Treatment Administration

All patients underwent surgical resection of the primary tumor followed by adjuvant radiotherapy with or without systemic chemotherapy. Patients in the chemoradiotherapy group received platinum–etoposide-based chemotherapy consisting of cisplatin administered either at a dose of 20 mg/m^2^ on days 1 through 5 or 75 mg/m^2^ on day 1, or carboplatin administered at an area under the curve (AUC) of 5, in combination with etoposide at a dose of 100 mg/m^2^ on days 1 through 3. Chemotherapy cycles were administered every 21 days for a total of four to six cycles, with the first two cycles delivered concurrently with radiotherapy. Adjuvant radiotherapy was administered to the primary tumor bed and regional lymphatic drainage areas using either three-dimensional conformal radiotherapy (3D-CRT) or intensity-modulated radiotherapy (IMRT). The prescribed radiotherapy dose generally ranged between 45 and 50 Gy delivered in 25 fractions, with an additional sequential boost of 9–10 Gy delivered to areas of residual disease or regions considered to be at particularly high risk of recurrence when clinically indicated.

### 2.5. Statistical Analysis

Categorical variables were summarized as frequencies and percentages, whereas continuous variables were summarized as means with standard deviations or medians with interquartile ranges depending on data distribution. Comparisons between treatment groups were performed using the chi-square test or Fisher’s exact test for categorical variables and the Student’s *t*-test or Mann–Whitney U test for continuous variables as appropriate. Survival outcomes, including overall survival (OS) and disease-free survival (DFS), were estimated using the Kaplan–Meier method, and differences between treatment groups were assessed using the log-rank test. Multivariable Cox proportional hazard regression models were used to evaluate the association between the addition of chemotherapy and survival outcomes while adjusting for potential confounding variables including age, sex, disease stage, tumor location, and sun-exposure status. A two-sided *p*-value of less than 0.05 was considered statistically significant. All statistical analyses were performed using IBM SPSS Statistics (version 29) (IBM Corp., Armonk, NY, USA).

## 3. Results

A total of 103 patients were included in this study. Of these, 26 patients received radiotherapy alone, while 77 patients received combined chemotherapy and radiotherapy. Sex distribution was slightly skewed toward males overall, with 67 males (65.0%) and 36 females (35.0%). In the radiotherapy-only group, sex was equally distributed (13 males, 13 females; 50.0% each), whereas in the combined therapy group, males predominated (54 males, 70.1%; 23 females, 29.9%). The mean age at diagnosis for the entire cohort was 70.8 ± 13.9 years, with a median age of 72 years (range, 36.0–95.6). Patients in the radiotherapy-only group were older on average (mean 76.2 ± 12.1 years; median 79.2 years; range, 51.8–95.6), compared to the combined therapy group (mean 68.9 ± 14.2 years; median 70.2 years; range, 36.0–95.1).

Regarding TNM stage, the majority of patients had stage IIIB disease (47 patients, 45.6%), followed by stage IIIA (41 patients, 39.8%) and stage IIB (15 patients, 14.6%). Patients receiving radiotherapy alone were more likely to have stage IIIA (15 patients, 57.7%) or stage IIB (six patients, 23.1%), whereas stage IIIB predominated in the combined therapy group (42 patients, 54.5%). Tumor location was variable. The most common sites were the head/face/lip (27 patients, 26.2%) and trunk (20 patients, 19.4%). Upper extremity tumors accounted for 21 patients (20.4%), lower extremity tumors for 14 patients (13.6%), groin/axilla for 16 patients (15.5%), and neck for five patients (4.9%). Distribution was generally similar between the treatment groups, with slight differences (e.g., groin/axilla tumors were more common in the combined therapy group, 13 vs. 3 in radiotherapy-only), (Table 1).

When tumor location was analyzed based on sun-exposure status, patients with non-sun-exposed tumors showed longer survival outcomes. Overall, 60 of 103 tumors (58.3%) were located in sun-exposed sites, including the head/neck/lip (30 patients, 29.1%), arm/hand (20 patients, 19.4%), and leg (10 patients, 9.7%). In the radiotherapy-only group, 16 of 26 patients (61.5%) had tumors in sun-exposed sites, while in the chemotherapy plus radiotherapy group, 44 of 77 patients (57.1%) had tumors located in sun-exposed areas.

Non-sun-exposed sites accounted for 43 patients (41.7%) overall. These included chest/trunk/back/abdomen (eight patients, 7.8%), buttock/groin/axilla (30 patients, 29.1%), and thigh/hip (five patients, 4.9%). Among patients receiving radiotherapy alone, 10 of 26 tumors (38.5%) were in non-sun-exposed areas, whereas 33 of 77 tumors (42.9%) in the combined therapy group were non-sun-exposed, (Table 2).

**Figure 2:**
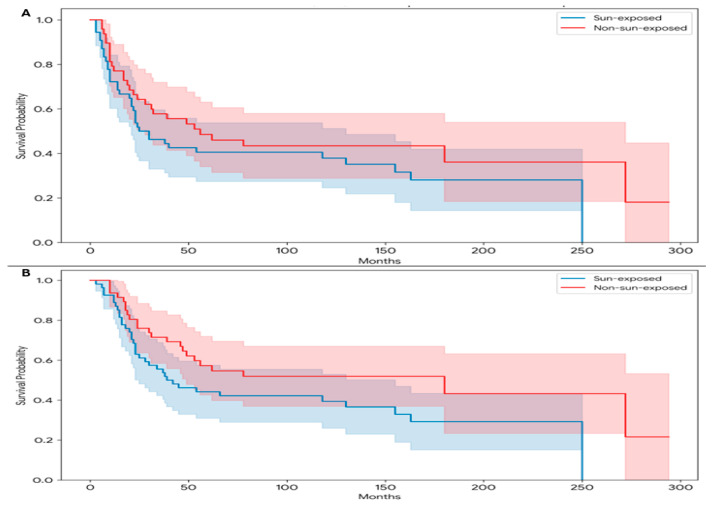
Kaplan–Meier survival curves according to tumor sun-exposure status. (**A**) Disease-free survival (DFS) according to tumor location. Patients with tumors located at non-sun-exposed sites (red line) had a median DFS of 57 months (95% CI, 32–180) compared with 42 months (95% CI, 22–120) for patients with tumors at sun-exposed sites (blue line) (log-rank *p* = 0.15; HR = 0.76, 95% CI, 0.52–1.11). (**B**) Overall survival (OS) according to tumor location. Patients with tumors located at non-sun-exposed sites (red line) had a median OS of 179 months (95% CI, 52–272) compared with 109 months (95% CI, 40–160) for patients with tumors at sun-exposed sites (blue line) (log-rank *p* = 0.054; HR = 0.68, 95% CI, 0.46–1.01).

When comparing the DFS according to sun exposure vs non-sun exposure, the results showed that survival outcomes were further analyzed according to tumor sun-exposure classification and treatment modality. Patients with tumors located at non-sun-exposed sites demonstrated a numerically longer median DFS (57 months) compared with the sun-exposed group (42 months), without statistical significance (log-rank *p* = 0.15). The median OS of patients with non-sun-exposed sites compared with those with tumors at sun-exposed sites (179 months vs. 109 months, respectively) was similar, although this difference did not reach statistical significance (log-rank *p* = 0.054), (Figure 2).

**Figure 3:**
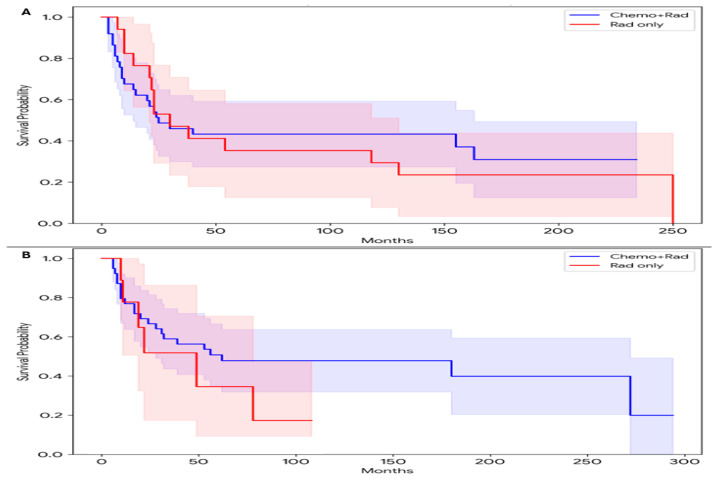
Disease-free survival (DFS) according to tumor sun-exposure status and treatment modality. (**A**) Kaplan–Meier curve for DFS among patients with tumors located at sun-exposed sites. Patients treated with chemotherapy plus radiotherapy (blue line) had a median DFS of 42 months (95% CI, 23–155) compared with 34 months (95% CI, 22–118) for patients treated with radiotherapy alone (red line) (log-rank *p* = 0.051; HR = 0.69, 95% CI, 0.48–1.00). (**B**) Kaplan–Meier curve for DFS among patients with tumors located at non-sun-exposed sites. Patients treated with chemotherapy plus radiotherapy (blue line) had a median DFS of 49 months (95% CI, 28–108) compared with 78 months (95% CI, 22–180) for patients treated with radiotherapy alone (red line) (log-rank *p* = 0.078; HR = 0.62, 95% CI, 0.36–1.06).

For DFS, among patients with sun-exposed tumors, the median DFS was 42 months in the chemotherapy plus radiotherapy group compared with 34 months in the radiotherapy-only group, although this difference was not statistically significant (log-rank *p* = 0.051). Among patients with non-sun-exposed tumors, the median DFS was 49 months in patients treated with combined chemotherapy and radiotherapy and 78 months in those treated with radiotherapy alone, with no statistically significant difference (*p* = 0.078), (Figure 3).

**Figure 4:**
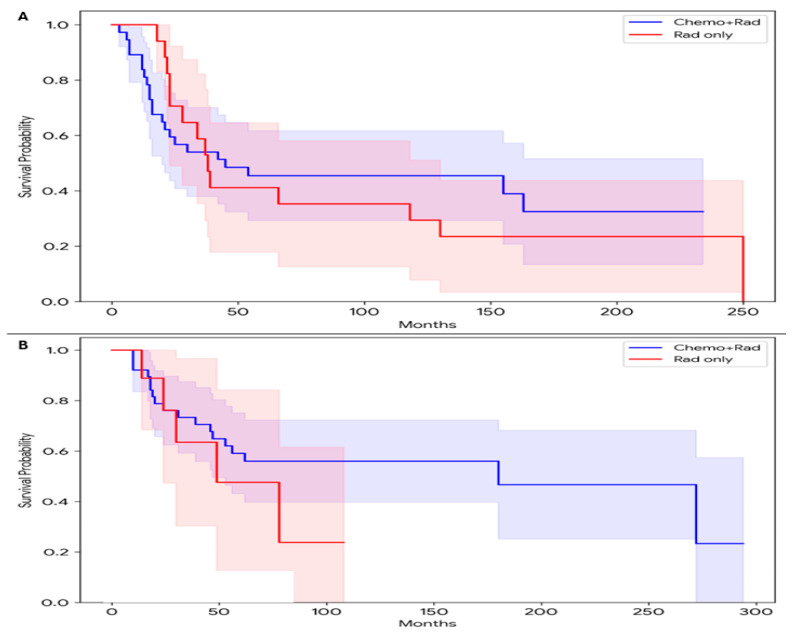
Overall survival (OS) according to tumor sun-exposure status and treatment modality. (**A**) Kaplan–Meier curve for OS among patients with tumors located at sun-exposed sites. Patients treated with chemotherapy plus radiotherapy (blue line) had a median OS of 128 months (95% CI, 38–234) compared with 98 months (95% CI, 22–130) for patients treated with radiotherapy alone (red line) (log-rank *p* = 0.08; HR = 0.71, 95% CI, 0.48–1.05). (**B**) Kaplan–Meier curve for OS among patients with tumors located at non-sun-exposed sites. Patients treated with chemotherapy plus radiotherapy (blue line) had a median OS of 178 months (95% CI, 45–272) compared with 56 months (95% CI, 26–64) for patients treated with radiotherapy alone (red line) (log-rank *p* = 0.054; HR = 0.53, 95% CI, 0.28–1.01).

For OS, patients with sun-exposed tumors demonstrated a median OS of 128 months in the chemoradiotherapy group compared with 98 months in the radiotherapy-only group, without a statistically significant difference (*p* = 0.08). Among patients with non-sun-exposed tumors, the median OS was 178 months in the combined treatment group and 56 months in the radiotherapy-only group, showing a statistical trend for reaching significance (log-rank *p* = 0.054), (Figure 4).

A multivariate analysis was performed to evaluate the effect of treatment modality on 5-year OS and 5-year DFS across different anatomical regions. In patients with head and neck tumors, treatment with combined chemotherapy and radiotherapy was associated with improved survival outcomes compared with radiotherapy alone. The 5-year OS rate was 50.0% in the chemoradiotherapy group compared with 30.0% in the radiotherapy-only group, demonstrating a statistically significant difference (*p* = 0.041). Similarly, the 5-year DFS rate was 46.2% in patients treated with combined therapy versus 30.0% in those receiving radiotherapy alone (*p* = 0.038). Among patients with tumors located at the groin and trunk, the 5-year OS was 55.7% in the chemoradiotherapy group and 47.6% in the radiotherapy-only group, without a statistically significant difference (*p* = 0.124). The 5-year DFS in this region was 48.4% with combined therapy and 34.6% with radiotherapy alone, also without statistical significance (*p* = 0.181).

For tumors arising in the extremities, survival outcomes were comparable between treatment groups. The 5-year OS rate was 46.6% in patients receiving chemoradiotherapy compared with 57.1% in those treated with radiotherapy alone (*p* = 0.051). The 5-year DFS rate was 46.7% in the combined therapy group, with no significant difference observed compared with radiotherapy alone (*p* = 0.064), (Table 3).

In the chemoradiotherapy group, OS was evaluated according to tumor exposure category and sex. Among patients with sun-exposed tumors, no statistically significant difference in OS was observed between males and females (*p* = 0.13). Similarly, within the non-sun-exposed tumor subgroup, there was also no significant difference in OS between sexes (*p* = 0.17).

## 4. Discussion

MCC is a rare and aggressive cutaneous neuroendocrine malignancy with a high propensity for recurrence and metastasis. Because of its aggressive biology and the historically high rates of relapse, adjuvant treatment strategies have often included radiotherapy with or without systemic chemotherapy. However, the role of adjuvant chemotherapy in localized or locally advanced MCC remains controversial. The present multicenter retrospective cohort study evaluated whether the addition of platinum–etoposide chemotherapy to adjuvant radiotherapy improves outcomes in patients with high-risk MCC (stage IIB–III).

In the current cohort of 103 patients, 77 patients (74.8%) received combined chemoradiotherapy and 26 patients (25.2%) received radiotherapy alone following surgical resection. Overall survival and disease-free survival analyses demonstrated no statistically significant improvement associated with the addition of chemotherapy, either in the overall population or when stratified according to tumor site exposure. These findings are consistent with several previously published studies suggesting that adjuvant chemotherapy may not provide a meaningful survival benefit in resected high-risk MCC.

Historically, chemotherapy regimens used in MCC were extrapolated from the treatment of small cell lung cancer, typically involving platinum plus etoposide combinations. Early reports suggested high response rates in metastatic disease; however, these responses were often short-lived and rarely translated into improved long-term survival [14]. In a retrospective analysis from the Trans-Tasman Radiation Oncology Group (TROG 96:07) trial, chemoradiotherapy using carboplatin and etoposide demonstrated good locoregional control but did not significantly improve overall survival compared with historical controls [15].

Similarly, several retrospective population-based analyses have failed to demonstrate a clear survival benefit from adjuvant chemotherapy. For example, an analysis of the National Cancer Data Base including more than 4800 MCC patients reported that the use of adjuvant chemotherapy did not significantly improve overall survival compared with radiotherapy alone, despite being more frequently administered in younger and higher-risk patients [16]. In that study, the 5-year overall survival rate was approximately 40–45% regardless of chemotherapy use, suggesting limited benefit from systemic cytotoxic therapy in the adjuvant setting.

Our findings align with these observations. In the present study, survival outcomes were comparable between treatment groups despite a higher proportion of stage IIIB disease in the chemoradiotherapy group (54.5%) compared with the radiotherapy-only group (19.2%). This imbalance suggests that clinicians may have preferentially selected combined therapy for patients perceived to be at higher risk of recurrence, which is consistent with real-world treatment patterns reported in previous studies [17].

An additional finding of the current study relates to the potential prognostic impact of tumor location according to sun-exposure status. Tumors arising in non-sun-exposed sites demonstrated numerically longer survival outcomes, with a median DFS of 57 months compared with 42 months for sun-exposed tumors, and a median OS of 179 months versus 109 months, respectively. However, these differences did not reach statistical significance (DFS *p* = 0.15; OS *p* = 0.054), although the OS difference suggests a borderline trend toward improved survival in the non-sun-exposed group.

This observation may reflect the recognized heterogeneity of MCC. Two major pathogenic pathways have been described: Merkel cell polyomavirus (MCPyV)-associated tumors and UV-induced virus-negative tumors. Virus-negative MCC, which is more commonly associated with chronically sun-damaged skin, tends to exhibit a higher mutational burden and distinct molecular characteristics [18]. Some studies have suggested that MCPyV-positive tumors, which may arise in less sun-exposed areas, can demonstrate different clinical behavior and immune responses [19]. Although viral status was not available in our cohort, the survival differences observed between sun-exposed and non-sun-exposed sites may partially reflect these underlying biological differences.

When treatment effects were analyzed within exposure subgroups, no statistically significant benefit of adding chemotherapy was observed. Among patients with sun-exposed tumors, the median DFS was 42 months in the chemoradiotherapy group compared with 34 months in the radiotherapy-alone group, and the median OS was 128 months versus 98 months, respectively; however, these differences did not reach statistical significance (*p* = 0.051 for DFS and *p* = 0.08 for OS). In contrast, among patients with non-sun-exposed tumors, radiotherapy alone was associated with numerically similar or even longer survival outcomes compared with combined treatment, with a median DFS of 78 months versus 56 months and a median OS of 192 months versus 178 months, respectively, again without statistical significance (*p* = 0.078 for DFS and *p* = 0.054 for OS). These findings further support the conclusion that the addition of chemotherapy did not provide a consistent survival advantage, but in some cases it may be effective.

The impact of primary tumor location on outcomes in MCC remains an important and debated prognostic factor. In our study, we evaluated the influence of tumor location in a cohort limited to non-metastatic, high-risk MCC (stage IIB–III). Interestingly, while the prior literature has frequently reported worse outcomes for head and neck MCC, our findings suggest that the addition of adjuvant chemotherapy may mitigate some of this risk in selected high-risk patients. Previous studies, including cohorts that encompassed both non-metastatic and metastatic disease, have consistently demonstrated poorer outcomes for head and neck MCC. These findings have been attributed to several factors, including older patient age, anatomical complexity limiting surgical margins, and unique lymphatic drainage patterns associated with head and neck tumors. Achieving adequate surgical margins in cosmetically and functionally sensitive areas may be more challenging, potentially contributing to increased local recurrence and worse survival outcomes. Supporting this observation, an analysis of more than 7800 cases from the National Cancer Database demonstrated that head and neck tumors were associated with significantly increased mortality compared with tumors of the extremities [20]. The adjusted hazard ratios were 1.306 (95% CI: 1.190–1.433) for head and neck versus upper extremity tumors, and 1.319 (95% CI: 1.180–1.473) for head and neck versus lower extremity tumors. Similarly, another large analysis reported significantly lower survival for head and neck MCC compared with other primary tumor sites, with a five-year overall survival of 38.7% versus 47.3% (*p* < 0.001). Outcomes were particularly poor for scalp and neck primaries, with a five-year overall survival of 29.7% compared with 41.3% for other head and neck sites. Additional studies have further corroborated these findings, consistently demonstrating poorer outcomes for head and neck MCC compared with other anatomical locations [21,22].

However, it is important to interpret our findings in the context of the study population. Our cohort included only high-risk patients with stage IIB and stage III disease, and therefore most patients—particularly those with stage III disease—had lymph node involvement at diagnosis regardless of tumor location. This reduces the likelihood that earlier detection alone explains differences in outcomes between tumor sites. Moreover, management of head and neck MCC may differ from other anatomical locations, often involving multidisciplinary evaluation, closer follow-up, and treatment at specialized centers. These factors, together with the addition of systemic therapy, may influence outcomes and potentially explain the observed benefit associated with chemotherapy in this subgroup.

Nevertheless, given the retrospective nature of this study and the limited number of patients in subgroup analyses, these findings should be interpreted cautiously. The observed impact of tumor location and the potential benefit of adjuvant chemotherapy in head and neck MCC should therefore be considered hypothesis-generating and require confirmation in larger prospective studies.

Comparable findings have been reported in other retrospective cohorts. For example, a multicenter European study evaluating adjuvant treatment strategies in MCC found no improvement in disease-free or overall survival with chemotherapy, despite increased toxicity and treatment-related complications [23]. Similarly, a systematic review reported that adjuvant chemotherapy did not significantly reduce recurrence rates or improve survival, while potentially increasing treatment-related morbidity [24].

The therapeutic landscape of MCC has evolved significantly in recent years with the introduction of immune checkpoint inhibitors, which have demonstrated durable responses in advanced disease. Agents targeting the PD-1/PD-L1 pathway, such as pembrolizumab, nivolumab, and avelumab, have produced response rates of 50–60% in metastatic MCC, often with long-lasting disease control [25,26,27]. As a result, systemic cytotoxic chemotherapy is increasingly being replaced by immunotherapy in many clinical scenarios. The lack of clear benefit from adjuvant chemotherapy observed in our study further supports this shift in treatment paradigms.

The present study has several limitations inherent to its retrospective design. First, treatment allocation was not randomized, and selection bias likely influenced physician decision-making. Patients receiving radiotherapy alone were older on average (median age 79 vs. 70 years), which may reflect concerns regarding chemotherapy tolerance. Second, the relatively small sample size, particularly in subgroup analyses, limits the statistical power to detect modest survival differences. Third, information regarding Merkel cell polyomavirus status, immune markers, and treatment-related toxicity was not available, which may have provided additional insights into treatment response and tumor biology. While tumors in the head and neck region may be detected earlier due to cosmetic visibility, our cohort included only high-risk stage IIB and stage III disease, with most patients presenting with lymph node involvement. Nevertheless, unmeasured confounders such as referral patterns, differences in multidisciplinary management, and treatment center experience may have influenced outcomes. An additional limitation of our study is the lack of comprehensive treatment-related adverse event data. Due to the retrospective design and the inclusion of patients treated over a prolonged period beginning in 1985, toxicity information was not consistently documented across participating centers, resulting in incomplete and non-uniform adverse event reporting. Consequently, treatment-related toxicities could not be reliably assessed or systematically analyzed in the present study. In addition, cause-specific mortality data were not consistently available; therefore, overall survival was analyzed without differentiation between cancer-related and non-cancer-related deaths, which may introduce bias in the interpretation of survival outcomes in this elderly cohort.

Despite these limitations, this study represents one of the larger real-world cohorts examining adjuvant treatment strategies in high-risk MCC across multiple centers over an extended follow-up period. The inclusion of patients treated over several decades allowed for the assessment of long-term survival outcomes, with several patients demonstrating survival exceeding 10–15 years, reflecting the potential for durable disease control in selected individuals. In addition, subgroup analyses stratified by anatomical location and other clinical variables included relatively small patient numbers, reflecting the inherent rarity of Merkel cell carcinoma. As a result, these exploratory analyses are limited by reduced statistical power and a higher risk of type II error. Therefore, findings derived from these subgroup comparisons should be interpreted with caution. Nevertheless, given the very low incidence of MCC, such real-world multicenter data remain valuable for generating hypothesis-driven insights into disease behavior, potential site-specific differences, and treatment effects, which may guide future prospective studies.

In conclusion, the results of this multicenter retrospective study suggest that the addition of platinum–etoposide chemotherapy to adjuvant radiotherapy does not significantly improve disease-free or overall survival in patients with high-risk localized or locally advanced MCC. Tumor location according to sun exposure may have potential prognostic implications, although further research is required to clarify its biological significance. Future prospective studies incorporating molecular profiling and modern immunotherapeutic approaches may help refine adjuvant treatment strategies and identify patient subgroups most likely to benefit from systemic therapy.

## 5. Conclusions

In this multicenter retrospective cohort study of 103 patients with high-risk Merkel cell carcinoma, tumor location according to sun-exposure status appeared to influence survival outcomes, with non-sun-exposed tumors showing a trend toward improved disease-free and overall survival. The addition of adjuvant platinum–etoposide chemotherapy was not associated with a statistically significant survival benefit in the overall cohort, although favorable trends were observed in selected subgroups.

These findings suggest that tumor anatomical and biological context may be relevant in stratifying outcomes and treatment response in MCC. Future studies should focus on prospective validation of these observations and on integrating tumor biological characteristics with modern immunotherapy approaches to better define optimal adjuvant treatment strategies rather than abstracting all study findings. Although limited by the retrospective design and sample size, the observed survival trends support further investigation of sun-exposure status as a clinically relevant stratification factor in Merkel cell carcinoma and warrant prospective validation in larger cohorts, particularly in the era of immunotherapy.

## Figures and Tables

**Table 1:** Baseline patient characteristics.

Characteristic	Overall (*n* = 103)	Radiotherapy Only (*n* = 26)	Chemotherapy and Radiotherapy (*n* = 77)
**Sex**			
Male	67 (65.0%)	13 (50.0%)	54 (70.1%)
Female	36 (35.0%)	13 (50.0%)	23 (29.9%)
**Age at diagnosis (years)**			
Mean ± SD	70.8 ± 13.9	76.2 ± 12.1	68.9 ± 14.2
Median	72	79.2	70.2
Range	36.0–95.6	51.8–95.6	36.0–95.1
**TNM Stage**			
IIB	15 (14.6%)	6 (23.1%)	9 (11.7%)
IIIA	41 (39.8%)	15 (57.7%)	26 (33.8%)
IIIB	47 (45.6%)	5 (19.2%)	42 (54.5%)
**Tumor Location**			
Head/Face/Lip	27 (26.2%)	7 (26.9%)	20 (26.0%)
Upper extremity	21 (20.4%)	6 (23.1%)	15 (19.5%)
Lower extremity	14 (13.6%)	3 (11.5%)	11 (14.3%)
Trunk (back/chest/abdomen/buttock)	20 (19.4%)	6 (23.1%)	14 (18.2%)
Groin/Axilla	16 (15.5%)	3 (11.5%)	13 (16.9%)
Neck	5 (4.9%)	1 (3.8%)	4 (5.2%)

Abbreviations: *n*; number.

**Table 2:** Distribution of treatment types and tumor locations according to sun exposure sites.

Characteristic	Overall (*n* = 103)	Radiotherapy Only (*n* = 26)	Chemotherapy (*n* = 77)
**Tumor Location (Sun-exposure classification)**			
**Sun-exposed sites**			
Head/neck/lip	30 (29.1%)	7 (26.9%)	23 (29.9%)
Arm/hand	20 (19.4%)	6 (23.1%)	14 (18.2%)
Leg	10 (9.7%)	3 (11.5%)	7 (9.1%)
Total sun-exposed	60 (58.3%)	16 (61.5%)	44 (57.1%)
**Non-sun-exposed sites**			
Chest/trunk/back/abdomen	8 (7.8%)	3 (11.5%)	5 (6.5%)
Buttock/groin/axilla	30 (29.1%)	4 (15.4%)	26 (33.8%)
Thigh/hip	5 (4.9%)	3 (11.5%)	2 (2.6%)
Total non-sun-exposed	43 (41.7%)	10 (38.5%)	33 (42.9%)

Abbreviations: *n*; number.

**Table 3:** Multivariate 5-year survival and efficacy table.

Anatomical Region	Treatment Type	5-Year OS (%)	*p*-Value (OS)	5-Year DFS (%)	*p*-Value (DFS)
Head & Neck	Chemo + Radiotherapy	50.00%	0.041	46.20%	0.038
	Radiotherapy Alone	30.00%	–	30.00%	–
Groin & Trunk	Chemo + Radiotherapy	55.70%	0.124	48.40%	0.181
	Radiotherapy Alone	47.60%	–	34.60%	–
Extremities	Chemo + Radiotherapy	46.60%	0.051	46.70%	0.064
	Radiotherapy Alone	57.10%	–	–	–

Abbreviations: OS, overall survival; DFS, disease-free survival; Chemo + Rad, chemotherapy plus radiotherapy.

## Data Availability

The data presented in this study are available on request from the corresponding author, because they contain individual patient information derived from medical records, and public sharing could compromise patient confidentiality. De-identified data may be made available by the corresponding author upon reasonable request and with approval from the relevant institutional review board, where applicable.

## Abbreviations

The following abbreviations are used in this manuscript:

MCC	Merkel Cell Carcinoma
DFS	Disease-Free Survival
OS	Overall Survival
TNM	Tumor, Node, Metastasis
CI	Confidence Interval
AJCC	American Joint Committee on Cancer
HR	Hazard Ratio
3D-CRT	Three-Dimensional Conformal Radiotherapy
IMRT	Intensity-Modulated Radiotherapy
Gy	Gray
AUC	Area Under the Curve
SD	Standard Deviation
IQR	Interquartile Range
UV	Ultraviolet
MCPyV	Merkel Cell Polyomavirus
PD-1	Programmed Cell Death Protein 1
PD-L1	Programmed Death-Ligand 1
SPSS	Statistical Package for the Social Sciences
N	Number of Patients
